# Molecular detection of virulence genes in *Staphylococcus aureus* isolated from wild pigeons (*Columba domestica livia*) in KwaZulu-Natal in South Africa

**DOI:** 10.1016/j.onehlt.2023.100656

**Published:** 2023-11-19

**Authors:** Trevor K. Wilson, Oliver T. Zishiri, Mohamed E. El Zowalaty

**Affiliations:** a Discipline of Genetics, School of Life Sciences, College of Agriculture, Engineering and Science, University of KwaZulu-Natal, Private Bag X54001, Durban 4000, South Africa; b Veterinary Medicine and Food Security Research Groups, Medical Laboratory Sciences Program, Faculty of Health Sciences, Abu Dhabi Women's Campus, Higher Colleges of Technology, Abu Dhabi 41012, United Arab Emirates

**Keywords:** *Staphylococcus aureus*, Virulence genes, Hospital, *LukS/F-PV*, *Hla*, *Hld*, *Spa*, *Sea*, *Musca domestica*, *Columba domestica livia*, Wild pigeons, Houseflies

## Abstract

The current study aimed to determine virulence determinants among *S. aureus* isolated from wild pigeons and houseflies around hospital areas in the Greater Durban area, South Africa. Following enrichment and bacterial growth, DNA extraction using the boiling method was performed. Overall, 57 out of 252 samples (22.6%) were positive for *S. aureus*. Six known virulence genes were tested, where five known virulence determinants were positive and none of the *S. aureus* isolates were positive to coagulase (*coa*) gene. The highest prevalence rates were found in the genes encoding haemolysins, with the *hla* and *hld* genes having 8 (14%) and 9 (15.8%) positive isolates respectively. The *sea*, *LukS/F-PV*, and *spa* genes had 5 (8.8%), 4 (7%), and 2 (3.5%) positive isolates respectively. These results demonstrated the detection of pathogenic *S. aureus* from hospital environment in Durban, South Africa which may account for the emergence staphylococcal infections. The findings of the present study highlights the significant role of wild pigeons and houseflies as potenital infectious disease vectors in a *One Health* context.

## 1 Introduction

*Staphylococcus aureus* (*S. aureus*) is one of the most common Gram-positive bacteria found across all environments and populations [1]. *S*. *aureus* is a non-motile, non-spore forming Gram-positive bacterium which can be found living commensally in approximately 20%-30% of the human population [1,2]. *S. aureus* is an opportunistic human pathogen causing both community and hospital acquired infections. It is responsible for several infections depending on the site of infection and the pathogenicity of the infecting strain [1,3]. Some examples of infections caused by *S. aureus* are infective endocarditis, bacteraemia, skin and soft tissue infections, pulmonary infections, and urinary tract infections [[4], [5], [6]]. The ability of *S*. *aureus* to form a biofilm is one of its mechanisms of survival and pathogenicity within the host, as the extracellular matrix of the biofilm holds the bacterial cells together, providing greater chances of survival within the host [7,8].

*S. aureus* has the capacity to infect a wide range of hosts, as well as being found to survive for long periods outside the host, in ubiquitous environments, which allows for greater opportunity for pathogen transmission and spillover events [9,10]. There are three main groups of *S. aureus,* specifically healthcare-associated, livestock-associated, and community-associated *S. aureus* [11,12]. Healthcare-associated *S. aureus* can spread from infected patients to healthcare workers through direct or close contact, and then from healthcare workers to other patients and staff members within the hospital setting [13]. Community-associated *S. aureus* can be found in most community settings, however it is most commonly found in areas favorable to bacterial growth, such as over-crowded, less-hygienic low-income areas [14]. Livestock-associated *S. aureus*  is capable of zoonotic transmission through direct contact with animals, and it is more likely isolated from those individuals who work in livestock farms and the agricultural industry [15]. Bacterial strains from all three groups have the capacity to be transmitted via a mechanical vector, such as houseflies [15].

*S. aureus* harbours an extensive arsenal of virulence factors which contribute to the pathogenicity of the bacterium. One such virulence factors is the alpha-haemolysin, an exotoxin often found in strains responsible for severe forms of skin and soft tissue staphylococcal infections [16]. The gene responsible for alpha-haemolysin is the *hla* gene while *hld* gene encodes delta-haemolysin, which has the capacity to cause membrane damage in many different cell types [17]. Another virulence factor is the Panton-Valentine Leucocidin, a cytotoxin known to destroy leukocytes and cause tissue necrosis [18]. This leucocidin is encoded by the *LukS/F-PV* gene. Three additional virulence factors specifically Staphylococcal Protein A (*spa*), Coagulase (*coa*), and Staphylococcal Enterotoxin A (*sea*) genes were investigated in the present study. The *spa* gene codes for Staphylococcal Protein A which is responsible for preventing the process of phagocytosis, allowing the pathogen to persist within the host [19]. Coagulase is also an important factor which prevents phagocytosis by promoting blood clotting in the host and causing fibrin to be produced, shielding the bacteria from phagocytosis [20]. The *coa* gene codes for the secretory protein Coagulase. Staphylococcal enterotoxin A has the capacity to cause staphylococcal food poisonings and is encoded by the *sea* gene [21].

The escalating numbers of staphylococcal infections around the world caused by *S. aureus* have spurred the search to detect pathogenic *S. aureus* in non-human hosts including livestock and wild birds. Very limited data are available in literature on the detection of *S. aureus* in pigeons in South Africa. The aim of the current study was to determine the prevalence of *S. aureus* isolated from fecal samples obtained from wild pigeons inhabiting the surrounding of a local hospital in Durban, KwaZulu-Natal, South Africa. In addition, we screened housefly samples randomly collected from different locations in the Greater Durban area for the presence of *S. aureus*. The prevalence of six different known virulence genes were tested using molecular methods using staphylococcal gene-specific PCR in *S. aureus* isolates in the present study.

## 2 Materials and methods

### 2.1 Ethical approval

The project was approved and permitted by the Animal Research Ethics Committee of the University of KwaZulu-Natal (Reference, AREC 071/017, and AREC 014/018). The field sampling protocols, sample collection, and the research were conducted in full compliance with Section 20 of the Animal Diseases Act of 1984 (Act No 35 of 1984) and were approved by the South African Department of Agriculture, Forestry and Fisheries DAFF (Section 20 approval Reference number 12/11/1/5).

### 2.2 Sample collection

Environmental fecal material and feather samples were randomly collected from pigeons outside a local hospital, in the Greater Durban area in KwaZulu-Natal Province during Winter (June to August) and Spring (September to November) in 2018 and 2022. Fresh fecal samples were obtained from the birds using sterile Amies agar transport swabs (Thermo Fisher Scientific, Waltham, MA, USA). Free pigeon feathers that appeared fresh and uncontaminated by pigeon feces were collected from the sampling site and were placed in screw cap tubes containing 10 mL of 0.1% peptone water.

**Fig. 1:**
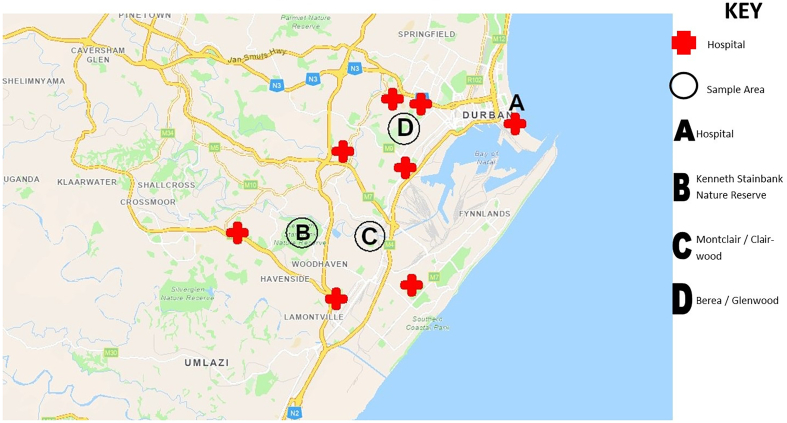
Geographic map showing the locations in the Greater Durban area in KwaZulu-Natal, South Africa where samples in the present study were collected from feral pigeons and houseflies. The map created using ArcGIS software.

Whole flies were collected using disposable fly traps which were placed around the sample sites (Fig. 1). The captured flies were then placed in tubes containing 70% ethanol. All samples were then transferred to the laboratory for further analysis.

### 2.3 Bacterial isolation

Environmental fecal samples obtained from pigeons were placed in 10 mL of 0.1% peptone water and incubated at 37 °C for 24 h. Thereafter, a volume of 100 μL of the culture was inoculated in 10 mL Brain Heart Infusion (BHI) broth (Thermo Fisher Scientific, Waltham, MA, USA) and incubated at 37 °C for another 24 h. The samples were streaked onto *S. aureus* ChromoSelect agar (Sigma-Aldrich, St. Louis, MO, USA) supplemented by egg yok tellurite emulsion. The plates were then placed in the incubator for 24 h at 37 °C. Those presumptive *S. aureus* colonies (brownish black colonies) were then kept as glycerol stocks until further analysis. Pigeon feathers were placed in tubes containing 0.1% peptone water and the feather was then discarded, and the peptone water medium was then streaked onto *S. aureus* ChromoSelect agar (Sigma-Aldrich, St. Louis, MO, USA) supplemented by egg yok tellurite emulsion.

The flies captured in the traps were homogenized using a sterile pestle and mortar and were placed into tubes containing BHI broth and incubated for 24 h at 37 °C. After. The sample was streaked onto *S. aureus* ChromoSelect agar supplemented with egg yolk tellurite emulsion (Sigma-Aldrich, St. Louis, MO, USA). Plates were placed in the incubator at 37 °C for 24 h and presumed *S. aureus* colonies were kept frozen as glycerol stocks for further analysis.

### 2.4 DNA extraction

Genomic DNA was extracted from each presumptively-identified *S. aureus* colonies using the standard boiling method as previously reported [22,23]. Presumptive *S. aureus* colonies were transferred from *S. aureus* ChromoSelect Agar into a tube containing 6 mL of Tryptone Soya Broth (Sigma-Aldrich, St. Louis, MO, USA) and placed in the incubator for 24 h at 37 °C. A volume of 400 μL of bacterial culture was placed into a sterile microcentrifuge tube and centrifuged for 15 min at 15000 × *g*. The supernatant was removed, and the pellet was reconstituted into 200 μL of molecular-grade, nuclease-free water, and centrifuged for 10 min at 15000*g*. Thereafter, removal of the supernatant was performed, and the pellet was reconstituted in 100 μL of TE buffer, where it was then boiled at 100 °C for 10 min. After boiling, the samples were centrifuged for 1 min at 15000 × *g*. The final supernatant was placed in a sterile microfuge tube and DNA concentration was measured using Nanorop.

### 2.5 Molecular detection of *S. aureus*

PCR primer sequences as shown in Table 1 [[25], [26], [27], [28], [29]] were purchased from Inqaba Biotec (Inqaba Biotec, Pretoria, South Africa). Successful PCR amplification of the species-specific thermonuclease (*nuc)* gene was used to confirm the presence of *S. aureus* in the sample. The PCR mixture consisted of 12.5 μl 2× DreamTaq Green PCR mastermix (Thermo Fisher Scientific, Waltham, MA, USA), 4 μl template DNA, 6.5 μl nuclease-free water, and 1 μl each of the forward and reverse primers (20 μM primer concentration), making the total volume in the tube 25 μl. Following the PCR amplification, gel electrophoresis was performed. A 1.5% (w/vol) agarose gel was used, and PCR products were loaded with 5 μl ethidium bromide (Thermo Fisher Scientific, Waltham, MA, USA). The electrophoresis was run for 45 min at 70 V while in Tris-borate-EDTA (pH 8.3, 1×). The molecular weight marker used was the 100 bp DNA ladder (Thermo Fisher Scientific, Waltham, MA, USA).

**Table 1:** Staphylococcal gene specific-primers used for polymerase chain reactions in the present study.

Gene	Protein	Primer	Primer Sequence (5′-3′)	Size (bp)	Reference
*nuc*	Thermonuclease	Primer 1 Primer 2	GCGATTGATGGTGATACGGTT AGCCAAGCCTTGACGAACTAAAGC	270	[24]
*LukS/FPV*	Panton-Valentine Leucocidin	PVL-1 NPVL-2	ATCATTAGGTAAAATGTCTGGACATGATCCA GCATCAASTGTATTGGATAGCAAAAGC	433	[25]
*hla*	Haemolysin	hlaF1 hlaF2	TTAGCCGAAAAACATCATTTC TTATTCCCGACGAAATTCCAA	960	[26]
*hld*		HLD-1 HLD-2	AAGAATTTTTATCTTAATTAAGGAAGGAGTG TTAGTGAATTTGTTCACTGTGTCGA	111	[25]
*spa*	*S. aureus* Protein A	Primer 1 Primer 2	CAAGCACCAAAAGAGGAA CACCAGGTTTAACGACAT	320	[27]
*coa*	Coagulase	COAG2 COAG3	CGAGACCAAGATTCAACAAG AAAGAAAACCACTCACATCA	730	[28]
*sea*	Enterotoxin A	Primer 1 Primer 2	GCAGGGAACAGCTTTAGGC GTTCTGTAGAAGTATGAAACACG	521	[29]

### 2.6 Molecular identification of *S. aureus* virulence genes

The detection of six known virulence genes was performed using PCR as previously reported [26]. Virulence genes including the *S. aureus* protein A (*spa)*, Panton-Valentine Leucocidin (*LukS/FPV)*, haemolysins (*hla* and *hld)*, coagulase (*coa)*, and enterotoxin A (*sea*) were screened in DNA samples extracted from *S. aureus* isolates.

### 2.7 Statistical analysis

To determine whether a significant relationship was present between the sample type collected / season of collection, and the resistance genes present, Pearson's Chi-Square Test and Fisher's Exact test were performed using the software program SPSS version 28 (IBM SPSS Statistics). A relationship was deemed significant if the *p* value was <0.05. Association between the presence/absence of one gene to another was assessed via Pearson's Correlation.

## 3 Results

Two hundred and fifty-two samples were collected during the Winter and Spring months, between June, 2018 to October 2022. A total of 88 samples were collected from pigeon feces, 62 from pigeon feathers, and 102 samples were collected from houseflies. All feral pigeon samples were collected from the pigeon population living within the grounds of a local public hospital (Location A) in Durban, KwaZulu-Natal Province, South Africa. A total of 12 fly samples were collected from location B, 50 samples were collected from location C, and 40 samples were from location D. A total of 123 samples were collected during the Winter months (June, July, and August) while 129 samples came during the Spring (September, October, and November) months.

It was found that 57 (22.62%) out of 252 samples tested were positive for the *S. aureus nuc* gene. Of these 57 positive samples, 29 were collected during the Winter months, while the remaining 28 were collected during Spring. It was found that 17 fecal samples and 13 feather samples from feral pigeons, and 27 samples from houseflies were positive for *nuc* gene of *S. aureus.*

**Fig. 2:**
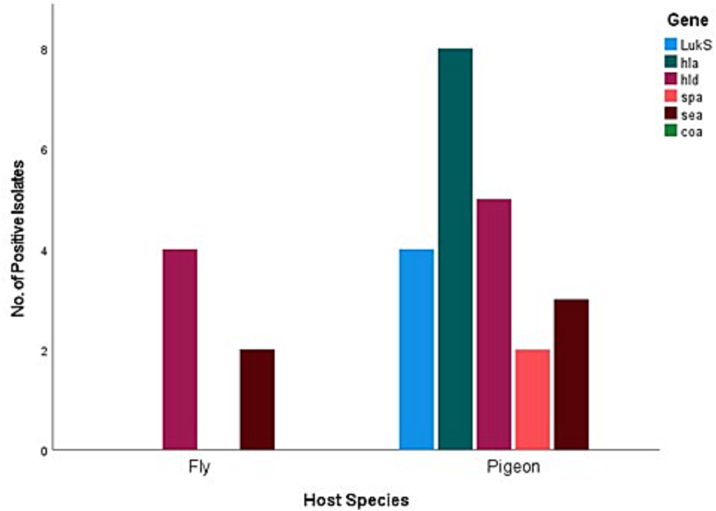
Different virulence genes identified in *Staphylococcus aureus* isolated from wild pigeons and houseflies. The figure shows that isolates from pigeons had more virulence genes than those from houseflies.

**Fig. 3:**
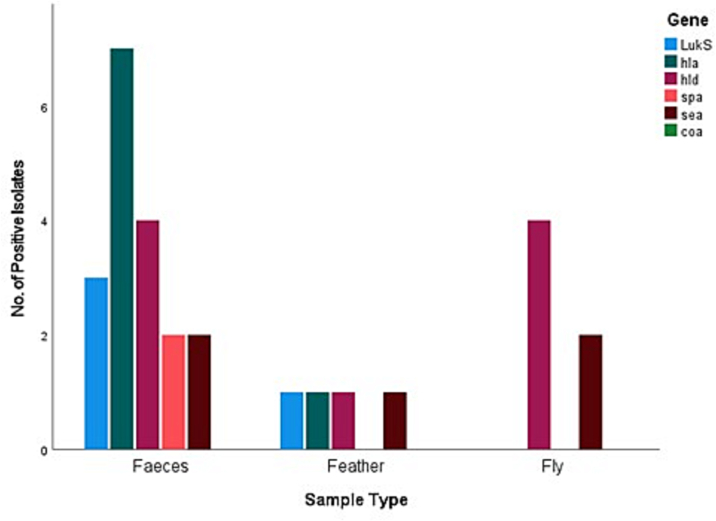
Different virulence genes identified in *S. aureus* isolated from wild pigeons and houseflies. The figure shows higher prevalence rates of detection of the tested virulence genes in *S. aureus* isolated from fecal samples than in isolates obtained from feather samples of wild pigeons.

**Fig. 4:**
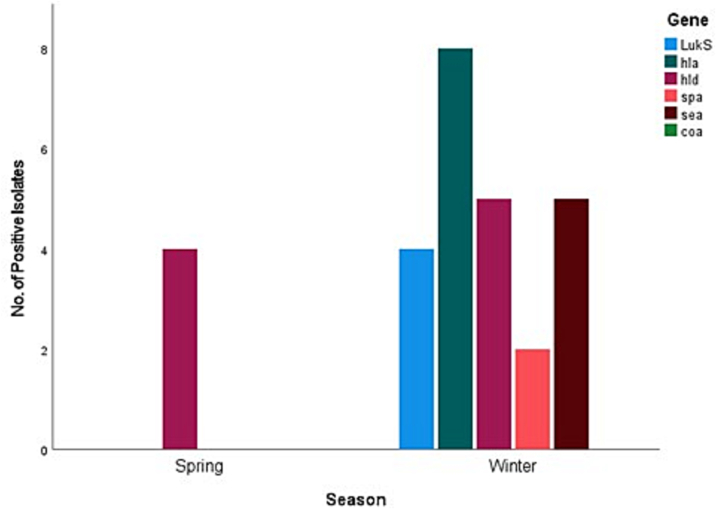
Different virulence genes identified in *S. aureus* isolated from wild pigeons and houseflies in the winter and spring. The figure shows higher prevalence rates of detection of the tested virulence genes in *S. aureus* isolated from samples collected in the winter than in samples collected in the spring. The figure also shows that *hld* was the only virulence gene detected in *S. aureus* isolated from samples collected in the spring.

**Fig. 5:**
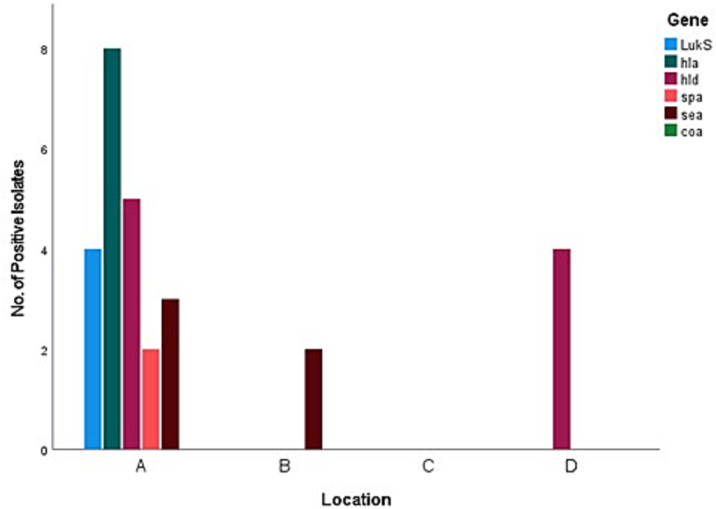
Detection of different virulence genes in *S. aureus* isolated from samples from houseflies captured from locations A, B, C and D in the Greater Durban area in South Africa. The figure shows higher prevalence rates of detection of the tested virulence genes in *S. aureus* isolated from samples collected from location A than in samples collected from locations B, C and D.

All the 57 samples were positive for five virulence genes but none of the 57 *S. aureus* isolates were positive for the *coa* gene in the present study. The most common virulence genes detected were the haemolysin genes, with *hla* and *hld* returning eight (14%) and nine (15.8%) positive isolates respectively, followed by *sea* with five (8.8%), *LukS/FPV* with four (7%), and finally *spa* with two (3.5%). Six (10.52%) of the 57 positive *S. aureus* isolates were positive for two or more virulence genes. Fig. 2, Fig. 3, Fig. 4, Fig. 5 represent the percentage of each virulence gene detected in *S. aureus* isolates from different host species (Fig. 2), sample type (Fig. 3), season of sample collection (Fig. 4), and the location of sample collection (Fig. 5).

Performance of Pearson's Chi Square and Fisher's Exact test was conducted to learn if there was a statistically significant (*p* < 0.05) relationship between the virulence genes detected and the host species, sample type, season, sample location (Table 2). Analysis revealed that the *hla* gene showed a statistically significant (p < 0.05) relationship between sample type, season, and host species from which *S. aureus* was isolated, but not the sampling location. The *sea* gene had a significant relationship with the sampling location of the isolate. All other relationships were found insignificant. Pearson's Correlation analysis revealed significant correlations between the *LukS/F-PV* and *sea* genes (0.4), along with significant correlation between the *spa* and *sea* genes (0.278) (Table 3).

**Table 2:** Probability values for Fisher's Exact and Pearson's Chi-Square tests performed to test the relationship between the virulence genes present and the sample type, season, location, and host species.

Virulence Gene	Sample Type		Season		Location		Host Species	
	*P*-Values							
	Pearson's Chi Square	Fisher's Exact	Pearson's Chi Square	Fisher's Exact	Pearson's Chi Square	Fisher's Exact	Pearson's Chi Square	Fisher's Exact
*LukS/FPV*	0.103	0.095	0.112	0.112	0.335	0.409	0.118	0.118
*hla*	<0.001⁎	<0.001⁎	0.004⁎	0.004⁎	0.059	0.057	0.006⁎	0.006⁎
*hld*	0.625	0.726	1	1	1	1	1	1
*spa*	0.148	0.148	0.491	0.491	0.592	0.592	0.495	0.495
*sea*	1	1	0.052	0.052	0.003⁎	0.006⁎	1	1

⁎ Significant figure to the 0.05 level.

**Table 3:** Pearson's Correlation values, with *p*-values in brackets.

Virulence Gene	Correlations				
	*LukS*	*hla*	*hld*	*spa*	*sea*
*LukS*	1	−0.111 (0.411)	0.069 (0.608)	−0.052 (0.699)	0.4⁎ (0.002)
*hla*	−0.111 (0.411)	1	0.102 (0.450)	0.197 (0.141)	−0.125 (0.353)
*hld*	0.069 (0.608)	0.102 (0.450)	1	0.179 (0.183)	−0.134 (0.319)
*spa*	−0.052 (0.699)	0.197 (0.141)	0.179 (0.183)	1	0.278⁎ (0.036)
*sea*	0.4⁎ (0.002)	−0.125 (0.353)	−0.134 (0.319)	0.278⁎ (0.036)	1

⁎ Correlation is significant at the 0.05 level (2-tailed).

## 4 Discussion

To the authors' knowledge, the present study is the first study to report the detection of *Staphylococcus aureus* and the virulence determinants in samples obtained from wild pigeons and houseflies in the Greater Durban area in KwaZulu-Natal, South Africa. *Staphylococcus aureus* is responsible for a vast array of infections. This ability is in part explained by the capability of the bacterium to express a significant number of virulence factors, with each factor being responsible for causing different adverse effects to the host [30]. It was reported that two constituent groups make up the virulence factors in *S. aureus*, those known as adherence factors, which are located on the bacterial surface, and those that are secreted (enterotoxins) [30]. In the current study, one adherence factor (Staphylococcal protein A, *spa*) and five enterotoxins (staphylococcal enterotoxin A, Panton-Valentine Leucocidin, alpha-haemolysin, delta-haemolysin, coagulase) genes were screened using primer-specific PCR to determine the prevalence of these factors in *S. aureus* isolated from pigeons and houseflies in Durban, South Africa. These genes were selected for analysis based on previous reports [9,22,31].

In the present study, all five virulence genes except the *coa* gene were detected in *S. aureus* isolated from pigeons and houseflies in the Greater Durban in South Africa. There was a low prevalence of the detected virulence genes, with the highest prevalence rate being 15.8% for the *hld* gene. The two genes encoding the alpha and delta-haemolysins had the highest prevalence among *S. aureus* isolates, which was similar to a previous study [9], where the highest prevalence of virulence genes was found in the *hld* gene, with 87.9% of isolates testing positive for this gene [9]. However, the difference between the present study and the study by Mkhize et al. [9] showing *hla* having the lowest frequency among the virulence genes tested in the study. This may be explained that pathogenicity caused by the haemolysin alpha protein is not common in *S. aureus* isolates in the current study as opposed to the previous study. The study by Mkhize et al. [9] did not include the *sea* gene but included the *LukS/F-PV* gene, which had a prevalence rate of 53.5%. The current study only found 7% prevalence rate for *LukS/F-PV* gene. Overall, when comparing the results of the current study to those reported in previous studies from KwaZulu-Natal Province, it shows decrease in the prevalence rates of almost all the virulence genes tested [9,22,31], with the exception of the prevalence rate of *LukS/F-PV* genes as compared to that reported by Dweba et al. [31], which reported similar results. The reasons for the low prevalence rates in the present study are unclear, but a possible explanation is the difference in the host species and the type of samples. The isolates reported in the previous studies were collected from different environments than to the isolates in the current study. In the present study, we collected samples from wild pigeons and houseflies in close proximity to a local public hospital where there appeared to be higher populations of houseflies. These sampling areas were likely more hygienic than the sampling areas reported in the previous studies, where the samples were collected from rural areas and farmlands [9,22,31], and there may be a chance for *S. aureus* populations to emerge in such environments. Another possible explanation for the decreased prevalence could be the enhanced countermeasure and increase in the hygienic level in the surrounding hospital areas during and after the COVID-19 pandemic which resulted in low incidence of staphylococcal and other bacterial infections.

It was reported that *Staphylococci* may be transmitted via mechanical vectors such as houseflies (*Musca domestica*) [15]. A gradual increase in the amount of litter in urban areas of South Africa as a result of poor service delivery was reported [16], which have contributed to the increase in the housefly populations. It was reported that houseflies are one of the significant vectors of infectious diseases of high risk to human and public health by harboring various pathogens both on their surface and within their gut [17]. The pathogens are not only limited to *S. aureus*, but several other hard to treat bacteria such as the ESKAPE pathogens (*Enterococcus faecium*, *Staphylococcus aureus*, *Klebsiella pneumoniae*, *Acinetobacter baumannii*, *Pseudomonas aeruginosa*, and *Enterobacter* species) which are the leading cause of nosocomial infections throughout the world [18,19]. Because of the close relationship of houseflies to human practices and their potential to be vectors for diseases caused through the spread of this bacterium, this makes the housefly a crucial target in identifying the spread of this bacterium. In the present study, samples were collected from pigeons and houseflies in close proximity to a local hospital where large populations of wild (rock) pigeons (*Columba livia*), are inhabiting the area and frequently fly in the surroundings of the hospital buildings and grounds. *S. aureus* was reported within hospital environments in KwaZulu-Natal province and isolates were detected from samples obtained from frequently touched sites such as beds, tables, and patient files in public hospitals in KwaZulu-Natal Province in South Africa [9].

It was reported that the prevalence rates of *LukS/F-PV*, *hla* and *sea* genes of *S. aureus* were 40%, 96.9%, and 4.6% respectively in a study from China [32]. Another study from Ethiopia reported prevalence rates of 93.75% and 87.5% for for *sea* and *hla* genes, respectively [33]. A study from the USA and Australia reported that *S. aureus* isolates were analysed for the presence of *LukS/F-PV*, *coa*, *hld*, *hla*, and *spa* genes, and high prevalence rates of 11% and 14% from the US and Australian isolates respectively were reported [34].

In the present study out of the five detected virulence genes, statistical analysis showed only that there is statistically significant (*p* < 0.05) relationships between sample type (<0.001), season (0.004), and host species (0.006) for *hla* gene. This may explain a significant relationship between pigeon populations and *hla* gene detection in the isolates collected from the birds. This highlights the possibility that *S. aureus* isolates obtained from pigeons inhabiting hospital grounds to produce alpha-haemolysin, making them more pathogenic than other strains found in the other areas sampled in this study. In a recent study in KwaZulu-Natal, it was reported that *sea*, *coa*, *LukS/F-PV*, and *spa* genes showed significant relationships with different factors such as location, host species, and sample material [31]. Obviously, an accurate comparison between the current study and the study by Dweba et al. [31] cannot be performed due to differences in the host species, location, and sample types.

In the present study, correlation analysis showed that *sea* gene was significantly correlated to *LukS/F-PV* and *spa* genes, both with positive correlations of 0.4 and 0.278 respectively. A similar significant positive correlation between *sea* and *LukS/F-PV* genes, although a weaker correlation was reported [31]. A significant correlation between *sea* and *spa* genes was not found in this previous study but was found between *LukS/F-PV* and *spa* genes [31]. The differences in correlations may be due to the different sampling locations, host species, and sample materials used between the two studies. The lower prevalence rates in the current study may be explained by the differences in correlations found.

In conclusion, to the authors' knowledge, the current study is the first to investigate and determine the detection of *S. aureus* in wild pigeon populations in close proximity to a local hospital in Durban, KwaZulu-Natal and its potential to be a reservoir of *S. aureus* and possibly other bacterial and viral pathogens which can be easily spread from hospital settings to the environmental surroundings. The detection of *S. aureus* isolates in feral pigeon populations highlights the zoonotic potential of wild pigeons to carry and transmit various pathogens (El Zowalaty, M.E., unpublished data). The findings of the present study demonstrated the carriage of *S. aureus* by houseflies and wild pigeon in hospital environment which may increases the potential for human exposure to pathogenic bacteria. An observation made during sample collection was that wild pigeon populations use of a watering fountain outside the hospital as pond for bathing, which is also used by the hospital visitors to rehydrate, which could result in the contamination of water leading to possible infections and outbreaks. The findings of the present study highlights the significant role of feral pigeons (*Columba domestica livia*) as potential vectors in disease transmission, illustrating the significance of the *One Health* interdisciplinary approach*.* It is recommended that proper hygiene and sanitation measures are urgently required to prevent the contamination of hospital settings and the surroundings with *S. aureus* from pigeon and housefly sources.

## Funding

This work was supported by the South African 10.13039/501100001321National Research Foundation through the Thuthuka Funding Instrument (grant number TTK170411226583).

## CRediT authorship contribution statement

**Trevor K. Wilson:** Methodology, Formal analysis, Visualization, Data curation, Investigation, Validation, Writing – original draft, Writing – review & editing. **Oliver T. Zishiri:** Conceptualization, Methodology, formal analysis, Investigation, Data curation, Supervision, Funding acquisition, Project administration, Resources, Software, Validation, Visualization, Writing – original draft, Writing – review & editing. **Mohamed E. El Zowalaty:** Conceptualization, Methodology, Investigation, Data curation, Formal analysis, Investigation, Visualization, Validation, Writing – original draft, Writing – review & editing, Project administration, Supervision, Funding acquisition.

## Declaration of Competing Interest

The authors declare that they have no known competing financial interests or personal relationships that could have appeared to influence the work reported in this manuscript.

## Data Availability

Data are available upon reasonable request.
